# Supplementary material for: The effect of body mass index reduction on intraocular pressure in a large prospective cohort of apparently healthy individuals in Israel
Source: PLoS One. 2023 May 17;18(5):e0285759. doi: 10.1371/journal.pone.0285759 (PMC10191308; doi:10.1371/journal.pone.0285759)
Supplement: Attachment — Submitted filename: Response to Reviewers.docx [file pone.0285759.s001.docx]

April 13, 2023

Dr. Emily Chenette

Editor-in-Chief

*PLOS ONE*

Dear Dr. Chenette,

Thank you very much for considering our manuscript entitled: “The effect of body mass index reduction on intraocular pressure in a large prospective cohort of apparently healthy individuals in Israel.”

We express our sincere appreciation to the editor and reviewers for providing valuable feedback that helped enhance the quality of our paper. Their constructive comments have enabled us to improve our manuscript significantly. We carefully considered each comment and provided a detailed response in the following pages.

We hope the revisions will fulfill the requirements for publication in *PLOS ONE.* Thank you very much for your time and consideration.

Sincerely,

Michael Waisbourd, MD

Director, Glaucoma Research Center

Clinical Associate Professor of Ophthalmology

Division of Ophthalmology

Tel-Aviv Medical Center

6 Weizmann Street Tel-Aviv, 64239, Israel

Email: [michaelwa@tlvmc.gov.il](mailto:michaelwa@tlvmc.gov.il)

Telephone: +972-3-6974165

**Dear Reviewers,**

Thank you for reviewing our manuscript. We truly appreciate your constructive comments and believe that your suggestions helped us improve our paper.

Please see the responses below, highlighted on the revised version of the manuscript.

**Journal Requirements**

1. Please ensure that your manuscript meets PLOS ONE's style requirements, including those for file naming.

**Reply:** Done.

2. Please provide additional details regarding participant consent. In the ethics statement in the Methods and online submission information, please ensure that you have specified (1) whether consent was informed and (2) what type you obtained (for instance, written or verbal, and if verbal, how it was documented and witnessed). If your study included minors, state whether you obtained consent from parents or guardians. If the need for consent was waived by the ethics committee, please include this information.
If you are reporting a retrospective study of medical records or archived samples, please ensure that you have discussed whether all data were fully anonymized before you accessed them and/or whether the IRB or ethics committee waived the requirement for informed consent. If patients provided informed written consent to have data from their medical records used in research, please include this information.

**Reply:** Done.

3. Please include your full ethics statement in the ‘Methods’ section of your manuscript file. In your statement, please include the full name of the IRB or ethics committee who approved or waived your study, as well as whether or not you obtained informed written or verbal consent. If consent was waived for your study, please include this information in your statement as well.

**Reply:** Done.

4. In your Data Availability statement, you have not specified where the minimal data set underlying the results described in your manuscript can be found. PLOS defines a study's minimal data set as the underlying data used to reach the conclusions drawn in the manuscript and any additional data required to replicate the reported study findings in their entirety. All PLOS journals require that the minimal data set be made fully available. For more information about our data policy, please see<http://journals.plos.org/plosone/s/data-availability>.

Upon re-submitting your revised manuscript, please upload your study’s minimal underlying data set as either Supporting Information files or to a stable, public repository and include the relevant URLs, DOIs, or accession numbers within your revised cover letter. For a list of acceptable repositories, please see <http://journals.plos.org/plosone/s/data-availability#loc-recommended-repositories>. Any potentially identifying patient information must be fully anonymized.

Important: If there are ethical or legal restrictions to sharing your data publicly, please explain these restrictions in detail. Please see our guidelines for more information on what we consider unacceptable restrictions to publicly sharing data: <http://journals.plos.org/plosone/s/data-availability#loc-unacceptable-data-access-restrictions>. Note that it is not acceptable for the authors to be the sole named individuals responsible for ensuring data access.

We will update your Data Availability statement to reflect the information you provide in your cover letter.

**Reply:** Upon request, the authors agree to share data as part of a signed data sharing agreement, based on our hospital’s data sharing policy.

**Additional Editor Comments:**

Though the sample size seems robust, it would be appreciated if the authors could comment on the power of study based on sample size and effect size.

**Reply**:

We included sample size calculation in the revised version of the manuscript

(Lines 108-110)

**Reviewer 1**

**Overall impression:**

In the manuscript entitled “The effect of body mass index reduction on intraocular pressure in a large prospective cohort of apparently healthy individuals in Israel“ it’s commendable that a large number of subjects were included as well a long period of examination, but the real goal of the examination remains unclear; is it: can we prevent glaucoma by losing body weight? Or?

**Reply:**

Thank you for your comment. We cannot conclude from our study that weight loss can prevent glaucoma, however our results clearly show that BMI reduction in associated with IOP reduction. We can speculate that this IOP reduction may reduce the risk for developing glaucoma, however the study was not design to answer this question. Based on your valuable comment, we revised our conclusions accordingly: “The implications on prevention of glaucoma in healthy individuals and possible protective effect in patients with glaucoma require further investigation.

(Lines 256-258)

**Major comments:**

1. The authors begin the introduction with the definition of glaucoma, but it is not clear

in the method section whether the subjects have glaucoma or some other eye disease.

**Reply**:

Our study included participants that attended periodic health screening examination as part of the Tel-Aviv Medical Center’s Executive Health Program. We included in our analysis individuals that underwent IOP measurements, regardless of the status of other eye diseases. We added this limitation as possible confounder under study limitations

(Lines 251-253)

2. In Methods is stated that ophthalmic evaluation included ocular history, visual

acuity and slit lamp; is it enough to confirm/rule out the existence of glaucoma or some other eye disease?

**Reply**:

Our comprehensive ophthalmic examination did identify eye diseases including glaucoma, however our analysis included all individuals who had IOP measurement, regardless of their final ocular diagnosis. We acknowledge this limitation in the revised manuscript as a possible confounding factor.

(Lines 251-253)

3. Methods: Which was inclusion/exclusion criteria for subjects who had IOP

measured?

**Reply**:

We added these inclusion and exclusion criteria to the methods. We included in our analysis all individuals who had at least one IOP and BMI measurements at their baseline visit.

(Lines 95-97)

4. The authors should explain why the subjects had the following examinations:

comprehensive laboratory and ancillary diagnostic tests, including blood chemistry

and metabolic profile, complete blood count, blood inflammatory markers, urine tests,

occult fecal blood test, prostate-specific antigen blood test (for men>40 years), cardiac

stress test, spirometry, audiometry, chest X-ray, and for women also gynecological

examination, including PAP test, physical breast examination, mammography, and

breast ultrasound.

**Reply**:

All participants in Tel-Aviv Medical Center's Executive Health Program received these tests by default in order to assess their overall health and detect potential health disorders or diseases. Individuals from this cohort who consented to enroll in the Tel Aviv Medical Center Inflammatory Survey (TAMCIS) and signed an informed consent form were included in our study. This information is noted under Study Methods. Outcomes of some of the above-mentioned tests were published elsewhere (In the past 3 years: PMID: 29491490, PMID: 30647453, PMID: 30880949, PMID: 31175686, PMID: 31864299, PMID: 33426569).

5. Methods: When was the second visit? What time period after the first visit?

**Reply**:

The mean (SD) time difference between the first and second visit was 1.95 (0.9) years. (Line 183)

6. Results: Which was baseline IOP range?

**Reply**: The baseline IOP range was between 5 and 31 mmHg. We added this information to the revised manuscript.

(Line 130)

7. Have any ophthalmological diseases appeared during the follow-up period?

**Reply**:

As mentioned under reply to comment 1, other eye diseases were not evaluated in the current analysis, and this is now mentioned as a study limitation in our revised discussion (Lines 251-253)

8. Why are laboratory indices indicated in Table 1?

**Reply**:

Since our study is addressing BMI and weight loss, especially among individuals with morbid obesity, we believe that adding baseline data on blood pressure and laboratory tests such as blood glucose level are relevant, since they may be directly related to obesity as part of the metabolic syndrome which includes obesity, blood pressure and diabetes.

9. What is clinical practice?

**Reply**:

We mention in our discussion: “Although our results were found in apparently healthy subjects, we can assume that BMI reduction will likely have a protective effect on patients with glaucoma, especially among morbidly obese individuals, since decrease of 1 mm Hg in IOP reduces the risk of glaucoma progression by 10%”.

(Lines 224-227)

**Reviewer 2**

**Overall impression:**
The authors present an interesting study on the association between body mass index and intraocular pressure and how weight loss leads to a decrease in intraocular pressure.

**Reply**:

Thank you for your comment.

**Major comments:**

1. The study covers a large number of patients, appropriate for a study of this type in

which patients have to be subdivided into different subgroups according to body mass index. However, the authors comment that the study exclusively includes apparently healthy individuals, but the methodology described does not seem to support this statement sufficiently. Pachymetry or examinations of the optic disc are not performed, so it cannot be ruled out that certain individuals have glaucoma (normotensive, or that by making only an IOP measurement, possible ocular hypertension has not been detected due to IOP fluctuations, thin pachymetries etc).

**Reply**:

Thank you for your valuable comment. Although participants did undergo a comprehensive eye examination, we did not exclude patients that were diagnosed with eye diseases (including glaucoma). This is an important limitation of our study, which is now mentioned under study limitations as a possible confounder.

(Lines 251-253)

2. Furthermore, it is not clear whether individuals with IOPs above normal after two

IOP measures were withdrawn from the study.

**Reply**:

Subjects with elevated IOP after 2 measurements were not excluded from the study cohort. We clarified this in our methods section: “IOP … of all eligible individuals was used for the current analysis”.

(Line 102)

3. It is commented that individuals who have weight loss have experienced a

decrease in IOP but it is not detailed whether this weight loss has been part of a standardized protocol to which all obese patients have been subjected, to carry out the descriptive study or has been an individual weight loss according to the criteria of each patient.

**Reply**:

Our results are based on observation of obese individuals who lost weight without any

standardized protocol as part of the study. This information was added to the revised

manuscript

(Line 94)

4. The discussion is a bit simple and poor, there are no comments on the glaucoma

association with all the blood parameters that have been determined and explained in the results and neither with the height of the patients and the smoking habit.

**Reply**:

We expanded the discussion based on your suggestions and now address association between glaucoma and parameters as well as smoking habits.

(Lines 230-232).

Lack of correlation between height and IOP is shown in Figure 3 (right panel) and is now also mentioned in the revised manuscript.

(Line 180)

5. The bibliography is somewhat outdated;

**Reply**:

The bibliography has been updated.

We added the following references:

a. Law SM, Lu X, Yu F, Tseng V, Law SK, Coleman AL. Cigarette smoking and glaucoma in the United States population. Eye (Lond). 2018;32(4):716-25

(Line 231)

b. Ng Yin Ling C, Lim SC, Jonas JB, Sabanayagam C. Obesity and risk of age-related eye diseases: a systematic review of prospective population-based studies. Int J Obes (Lond). 2021;45(9):1863-85

(Line 199)

c. Panon N, Luangsawang K, Rugaber C, Tongchit T, Thongsepee N, Cheaha D, et al. Correlation between body mass index and ocular parameters. Clin Ophthalmol. 2019;13:763-9

(Line 199)

d. Jabbehdari S, Chen JL, Vajaranant TS. Effect of dietary modification and antioxidant supplementation on intraocular pressure and open-angle glaucoma. Eur J Ophthalmol. 2021;31(4):1588-605

(Line 216)

e. Reddy A, Halenda K, Cromer P, Chen L, Butler J, Raed A, et al. The Association of Intraocular Pressure With Obesity and Cardiometabolic Risk in a Young Farmworker Population. J Glaucoma. 2021;30(1):24-31.

(Line 58)

6. The possible association between the measured parameters and IOP, and its possible clinical relevance, need to be further explained.

**Reply**:

As suggested, we discuss in the revised manuscript measured parameters including blood glucose levels, smoking status and systemic blood pressure and their clinical relevance to glaucoma and intraocular pressure

(Lines 237-242)

7. In the abstract, the conclusion part contains two sentences that are redundant since they say the same thing but with different words.

**Reply**:

The abstract conclusions were revised as suggested

(Lines 41-43)

8. The beta coefficient of Table 3 is not explained in the table legend nor in the text; the meaning and interpretation of it should be addressed in order to better understand the results.

**Reply**:

We expanded on the statistical meaning of the beta coefficient in the caption of Table 3. In addition, odds ratio was calculated for each parameter. We also discuss these findings in more detail in the results section

(Lines 164-168, Table 3)
